# {4-[(2,4-Dichloro­benzo­yloxy)meth­yl]-1-phenyl-1*H*-1,2,3-triazol-5-yl}methyl 2,4-dichloro­benzoate

**DOI:** 10.1107/S1600536811035550

**Published:** 2011-09-14

**Authors:** Dilmurot Ismatov, Umarkhon Azizov, Samat Talipov, Jamshid Ashurov

**Affiliations:** a Tashkent Chemical Technology Institute, A. Navoyi, 11, Tashkent, Uzbekistan; b Uzbekistan Scientific Research Pharmacological Chemistry Institute (named after A. Sultonov), Durmon yuli, 40, Uzbekistan; c Institute of Bioorganic Chemistry, Academy of Sciences of Uzbekistan, M. Ulugbek Str. 83, Tashkent, 100125 Uzbekistan

## Abstract

In the title molecule, C_24_H_15_Cl_4_N_3_O_4_, the triazole ring makes dihedral angles of 72.02 (12), 81.60 (12) and 73.82 (11)°, respectively, with the adjacent phenyl ring and the two dichloro­benzene rings. In the crystal, a weak C—H⋯N inter­action, a short Cl⋯Cl contact [3.307 (2) Å] and a π–π stacking inter­action [centroid–centroid distance = 3.568 (4) Å] are observed. An intra­molecular C—H⋯O inter­action is also present.

## Related literature

For the pharmacological activities of 1,2,3-triazole derivatives, see: Dzhuraev *et al.* (1990 ▶); Karimkulov *et al.* (1991 ▶); Zakirov *et al.* (2001 ▶). For a related structure, see: Jin *et al.* (2004 ▶). 
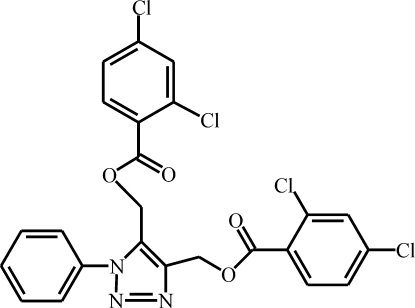


## Experimental

### 

#### Crystal data

C_24_H_15_Cl_4_N_3_O_4_
*M* *_r_* = 551.19
Monoclinic, 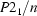

*a* = 8.908 (5) Å
*b* = 19.567 (5) Å
*c* = 13.908 (5) Å
β = 104.010 (5)°
*V* = 2352.1 (17) Å^3^
*Z* = 4
Cu *K*α radiation
μ = 4.91 mm^−1^
*T* = 293 K
0.6 × 0.4 × 0.3 mm

#### Data collection

Oxford Xcalibur Ruby diffractometer
Absorption correction: multi-scan (*CrysAlis PRO*; Oxford Diffraction, 2009 ▶) *T*_min_ = 0.050, *T*_max_ = 0.229
20081 measured reflections
4196 independent reflections
3370 reflections with *I* > 2σ(*I*)
*R*_int_ = 0.035

#### Refinement

*R*[*F* ^2^ > 2σ(*F* ^2^)] = 0.037
*wR*(*F* ^2^) = 0.107
*S* = 1.02
4196 reflections
317 parameters
H-atom parameters constrained
Δρ_max_ = 0.28 e Å^−3^
Δρ_min_ = −0.33 e Å^−3^

### 

Data collection: *CrysAlis PRO* (Oxford Diffraction, 2009 ▶); cell refinement: *CrysAlis PRO*; data reduction: *CrysAlis PRO*; program(s) used to solve structure: *SHELXS97* (Sheldrick, 2008 ▶); program(s) used to refine structure: *SHELXL97* (Sheldrick, 2008 ▶); molecular graphics: *XP* (Bruker, 1998 ▶); software used to prepare material for publication: *SHELXL97*.

## Supplementary Material

Crystal structure: contains datablock(s) I, global. DOI: 10.1107/S1600536811035550/is2762sup1.cif

Structure factors: contains datablock(s) I. DOI: 10.1107/S1600536811035550/is2762Isup2.hkl

Supplementary material file. DOI: 10.1107/S1600536811035550/is2762Isup3.cml

Additional supplementary materials:  crystallographic information; 3D view; checkCIF report

## Figures and Tables

**Table 1:** Hydrogen-bond geometry (Å, °)

*D*—H⋯*A*	*D*—H	H⋯*A*	*D*⋯*A*	*D*—H⋯*A*
C6—H6*B*⋯O4	0.97	2.49	3.280 (3)	139
C9—H9⋯N2^i^	0.93	2.58	3.288 (3)	134

Symmetry code: (i) 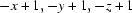.

**Fig. 1.**
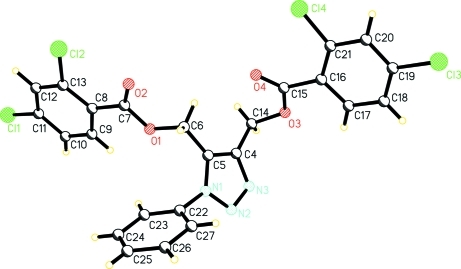
The molecular structure of the title compound, with the atom-numbering scheme.

**Fig. 2.**
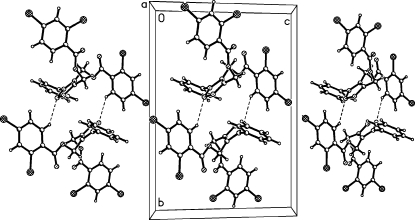
A packing diagram for the title compound. Dashed lines indicated C—H···N interactions.

## supplementary crystallographic information

### Comment

In last few decades, much attention has been paid to the synthesis of 1,2,3-triazole systems mainly due to their broad spectrum of pharmacological properties. 1,2,3-Triazole derivatives possess variety of pharmacological activities such as anti inflammatory, antiviral and antibacterial (Dzhuraev *et al.*, 1990; Karimkulov *et al.*, 1991; Zakirov *et al.*, 2001).

In the title compound, {4-[(2,4-dichlorobenzoyloxy)methyl]-1-phenyl-1*H*-1,2,3-triazol-5-yl}methyl 2,4-dichlorobenzoate, C_24_H_15_N_3_O_4_Cl_4_, the triazole ring (N1/N2/N3/C4/C5) is ideal planar with a greatest deviation of 0.0037 (12) Å (atom N3) from the mean plane and the benzyl rings of dichlorobenzoyloxy substituents (C8–C13 and C16–C21) are tilted out of this plane at 81.60 (12) and 73.82 (11)°, respectively. The dihedral angles between these benzyl rings and corresponding carboxylic fragments (O1/C7/O2 and O3/C15/O4) are 5.9 (4) and 26.9 (3)°, respectively. The dihedral angle between the triazole and phenyl (C22–C27) rings is 72.02 (12)°. The C5—N1 and C4—N2 bond lengths in the triazole ring are 1.352 (3) and 1.361 (3) Å, respectively. The values of these distances are shorter than the pertinent single bond length of 1.443 Å and are longer than the double bond length of 1.269 Å (Jin *et al.*, 2004).

An intermolecular Cl1···Cl1 (-*x*, 1 - *y*, 2 - *z*) contact and a π–π stacking interaction with a *Cg*1···*Cg*2 (*x* - 1/2, 1/2 - *y*, *z* + 1/2) distance of 3.568 (4) Å stabilize the crystal structure; *Cg*1 and *Cg*2 are the centroids of the C8–C13 and C16–C21 rings, respectively.

### Experimental

As a result of etherification 24.6 g (0.13 mole) of 2,4-dichlorbenzoic acid with 5.54 g (0.07 mole) of 2-butendiole-1, 4 refluxing for 2 h in benzene containing sulfuric acid as catalyst was got of 1,4-bis-(2,4-dichlorbenzoyloxy)-butene-2 [yield 22.55 g (82.2%), m.p. 364–365 K]. The reaction of obtaining bis-ester with 6.8 g (0.57 mole) phenylazide in 100 ml of toluene was carrying out within 7 h. Then the reaction mixture was cooled. The precipitate [1-phenyl-4,5-bis-(dichlorobenzoyloxymethyl)-1,2,3-triazole, yield 27.84 g (96.8%)] was collected by filtration and purified by recrystallization from ethanol (m.p. 383–384 K).

### Refinement

Aromatic (C—H = 0.93 Å) and methylene (C—H = 0.97 Å) H atoms were placed in geometrically calculated positions and refined using a riding model, with *U*_iso_(H) = 1.2*U*_eq_(C).

### Crystal data

**Table:**

C_24_H_15_Cl_4_N_3_O_4_	*F*(000) = 1120
*M**_r_* = 551.19	*D*_x_ = 1.557 Mg m^−^^3^
Monoclinic, *P*2_1_/*n*	Cu *K*α radiation, λ = 1.54184 Å
Hall symbol: -P 2yn	Cell parameters from 6999 reflections
*a* = 8.908 (5) Å	θ = 3.3–67.0°
*b* = 19.567 (5) Å	µ = 4.91 mm^−^^1^
*c* = 13.908 (5) Å	*T* = 293 K
β = 104.010 (5)°	Prismatic, colourless
*V* = 2352.1 (17) Å^3^	0.6 × 0.4 × 0.3 mm
*Z* = 4	

### Data collection

**Table:**

Oxford Xcalibur Ruby diffractometer	4196 independent reflections
Radiation source: fine-focus sealed tube	3370 reflections with *I* > 2σ(*I*)
graphite	*R*_int_ = 0.035
Detector resolution: 10.2576 pixels mm^-1^	θ_max_ = 66.9°, θ_min_ = 4.0°
ω scans	*h* = −9→10
Absorption correction: multi-scan (*CrysAlis PRO*; Oxford Diffraction, 2009)	*k* = −23→23
*T*_min_ = 0.050, *T*_max_ = 0.229	*l* = −16→16
20081 measured reflections	

### Refinement

**Table:**

Refinement on *F*^2^	Secondary atom site location: difference Fourier map
Least-squares matrix: full	Hydrogen site location: inferred from neighbouring sites
*R*[*F*^2^ > 2σ(*F*^2^)] = 0.037	H-atom parameters constrained
*wR*(*F*^2^) = 0.107	*w* = 1/[σ^2^(*F*_o_^2^) + (0.0642*P*)^2^ + 0.6051*P*] where *P* = (*F*_o_^2^ + 2*F*_c_^2^)/3
*S* = 1.02	(Δ/σ)_max_ = 0.001
4196 reflections	Δρ_max_ = 0.28 e Å^−^^3^
317 parameters	Δρ_min_ = −0.33 e Å^−^^3^
0 restraints	Extinction correction: *SHELXL97* (Sheldrick, 2008), Fc^*^=kFc[1+0.001xFc^2^λ^3^/sin(2θ)]^-1/4^
Primary atom site location: structure-invariant direct methods	Extinction coefficient: 0.00144 (16)

### Special details

**Table:**

Geometry. All e.s.d.'s (except the e.s.d. in the dihedral angle between two l.s. planes) are estimated using the full covariance matrix. The cell e.s.d.'s are taken into account individually in the estimation of e.s.d.'s in distances, angles and torsion angles; correlations between e.s.d.'s in cell parameters are only used when they are defined by crystal symmetry. An approximate (isotropic) treatment of cell e.s.d.'s is used for estimating e.s.d.'s involving l.s. planes.
Refinement. Refinement of *F*^2^ against ALL reflections. The weighted *R*-factor *wR* and goodness of fit *S* are based on *F*^2^, conventional *R*-factors *R* are based on *F*, with *F* set to zero for negative *F*^2^. The threshold expression of *F*^2^ > σ(*F*^2^) is used only for calculating *R*-factors(gt) *etc*. and is not relevant to the choice of reflections for refinement. *R*-factors based on *F*^2^ are statistically about twice as large as those based on *F*, and *R*- factors based on ALL data will be even larger.

### Fractional atomic coordinates and isotropic or equivalent isotropic displacement parameters (Å^2^)

**Table:**

	*x*	*y*	*z*	*U*_iso_*/*U*_eq_	
Cl1	0.10801 (9)	0.46153 (5)	0.93488 (6)	0.0854 (3)	
Cl2	0.19070 (9)	0.22022 (3)	0.77880 (5)	0.0670 (2)	
Cl3	1.25569 (8)	0.07713 (3)	0.26450 (5)	0.0597 (2)	
Cl4	1.01206 (10)	0.09158 (3)	0.57671 (5)	0.0710 (2)	
O1	0.44606 (18)	0.34288 (7)	0.59759 (11)	0.0417 (4)	
O2	0.3595 (2)	0.24058 (8)	0.62934 (13)	0.0562 (4)	
O3	0.98996 (17)	0.31279 (8)	0.52445 (12)	0.0459 (4)	
O4	0.8516 (2)	0.22445 (9)	0.55843 (14)	0.0592 (5)	
N1	0.52546 (19)	0.39978 (8)	0.40630 (12)	0.0363 (4)	
N2	0.6236 (2)	0.44090 (10)	0.37294 (15)	0.0471 (5)	
N3	0.7620 (2)	0.42975 (9)	0.42858 (15)	0.0470 (5)	
C4	0.7548 (2)	0.38112 (10)	0.49722 (15)	0.0391 (5)	
C5	0.6030 (2)	0.36183 (10)	0.48375 (14)	0.0354 (4)	
C6	0.5258 (3)	0.30865 (10)	0.53193 (16)	0.0404 (5)	
H6A	0.4526	0.2829	0.4820	0.048*	
H6B	0.6021	0.2772	0.5692	0.048*	
C7	0.3658 (2)	0.30065 (10)	0.64358 (14)	0.0342 (4)	
C8	0.2928 (2)	0.33971 (10)	0.71267 (14)	0.0344 (4)	
C9	0.3061 (3)	0.41067 (11)	0.71796 (17)	0.0430 (5)	
H9	0.3562	0.4331	0.6757	0.052*	
C10	0.2474 (3)	0.44875 (13)	0.78378 (18)	0.0523 (6)	
H10	0.2567	0.4961	0.7855	0.063*	
C11	0.1748 (3)	0.41542 (14)	0.84679 (17)	0.0529 (6)	
C12	0.1561 (3)	0.34554 (15)	0.84364 (17)	0.0525 (6)	
H12	0.1048	0.3238	0.8859	0.063*	
C13	0.2148 (2)	0.30801 (12)	0.77651 (15)	0.0420 (5)	
C14	0.8976 (3)	0.35859 (13)	0.56945 (18)	0.0502 (6)	
H14B	0.9590	0.3983	0.5957	0.060*	
H14A	0.8696	0.3354	0.6242	0.060*	
C15	0.9516 (2)	0.24628 (11)	0.52285 (15)	0.0405 (5)	
C16	1.0439 (2)	0.20499 (10)	0.46763 (15)	0.0364 (4)	
C17	1.1026 (2)	0.23534 (11)	0.39367 (16)	0.0401 (5)	
H17	1.0943	0.2824	0.3850	0.048*	
C18	1.1725 (3)	0.19760 (11)	0.33304 (16)	0.0422 (5)	
H18	1.2107	0.2188	0.2841	0.051*	
C19	1.1847 (2)	0.12779 (11)	0.34625 (16)	0.0413 (5)	
C20	1.1354 (3)	0.09624 (11)	0.42153 (17)	0.0460 (5)	
H20	1.1486	0.0494	0.4316	0.055*	
C21	1.0662 (3)	0.13477 (11)	0.48190 (16)	0.0421 (5)	
C22	0.3629 (2)	0.40125 (10)	0.35910 (15)	0.0372 (5)	
C23	0.2613 (3)	0.43117 (11)	0.40706 (18)	0.0468 (5)	
H23	0.2967	0.4500	0.4699	0.056*	
C24	0.1058 (3)	0.43268 (13)	0.3600 (2)	0.0605 (7)	
H24	0.0357	0.4525	0.3914	0.073*	
C25	0.0543 (3)	0.40524 (14)	0.2673 (3)	0.0658 (8)	
H25	−0.0507	0.4062	0.2365	0.079*	
C26	0.1570 (3)	0.37622 (14)	0.2194 (2)	0.0636 (7)	
H26	0.1213	0.3583	0.1561	0.076*	
C27	0.3123 (3)	0.37379 (12)	0.26507 (17)	0.0487 (5)	
H27	0.3821	0.3540	0.2333	0.058*	

### Atomic displacement parameters (Å^2^)

**Table:**

	*U*^11^	*U*^22^	*U*^33^	*U*^12^	*U*^13^	*U*^23^
Cl1	0.0745 (5)	0.1223 (7)	0.0652 (4)	0.0175 (5)	0.0282 (4)	−0.0319 (4)
Cl2	0.0858 (5)	0.0543 (4)	0.0696 (4)	−0.0209 (3)	0.0358 (4)	0.0128 (3)
Cl3	0.0716 (4)	0.0545 (4)	0.0595 (4)	0.0131 (3)	0.0285 (3)	−0.0055 (3)
Cl4	0.1151 (6)	0.0443 (3)	0.0688 (4)	0.0069 (3)	0.0518 (4)	0.0151 (3)
O1	0.0529 (9)	0.0339 (7)	0.0469 (8)	−0.0018 (6)	0.0285 (7)	0.0012 (6)
O2	0.0788 (12)	0.0353 (8)	0.0641 (10)	−0.0067 (8)	0.0359 (9)	−0.0011 (7)
O3	0.0415 (9)	0.0389 (8)	0.0590 (9)	0.0030 (6)	0.0157 (7)	−0.0087 (7)
O4	0.0621 (11)	0.0522 (10)	0.0737 (11)	0.0043 (8)	0.0368 (10)	0.0067 (8)
N1	0.0382 (9)	0.0336 (9)	0.0429 (9)	0.0017 (7)	0.0210 (8)	0.0021 (7)
N2	0.0469 (11)	0.0420 (10)	0.0600 (12)	−0.0006 (8)	0.0275 (10)	0.0082 (9)
N3	0.0428 (11)	0.0422 (10)	0.0624 (12)	−0.0025 (8)	0.0251 (10)	0.0002 (9)
C4	0.0429 (12)	0.0335 (10)	0.0450 (11)	0.0019 (9)	0.0184 (9)	−0.0092 (9)
C5	0.0434 (12)	0.0314 (10)	0.0362 (10)	0.0045 (8)	0.0188 (9)	−0.0035 (8)
C6	0.0493 (13)	0.0344 (10)	0.0429 (11)	0.0042 (9)	0.0218 (10)	0.0024 (8)
C7	0.0343 (11)	0.0352 (11)	0.0325 (10)	−0.0021 (8)	0.0069 (8)	0.0056 (8)
C8	0.0314 (10)	0.0396 (10)	0.0319 (10)	0.0001 (8)	0.0069 (8)	0.0052 (8)
C9	0.0437 (12)	0.0412 (11)	0.0470 (12)	0.0023 (9)	0.0163 (10)	0.0047 (9)
C10	0.0534 (14)	0.0481 (13)	0.0569 (14)	0.0078 (11)	0.0165 (12)	−0.0056 (11)
C11	0.0427 (13)	0.0741 (17)	0.0420 (12)	0.0121 (12)	0.0104 (10)	−0.0107 (11)
C12	0.0432 (13)	0.0786 (18)	0.0386 (12)	0.0023 (12)	0.0157 (10)	0.0093 (11)
C13	0.0383 (12)	0.0498 (12)	0.0377 (11)	−0.0021 (10)	0.0091 (9)	0.0084 (9)
C14	0.0486 (13)	0.0458 (12)	0.0556 (13)	0.0046 (10)	0.0115 (11)	−0.0160 (10)
C15	0.0397 (12)	0.0409 (11)	0.0381 (10)	0.0043 (9)	0.0040 (9)	0.0019 (9)
C16	0.0339 (11)	0.0354 (10)	0.0382 (10)	0.0005 (8)	0.0052 (8)	−0.0002 (8)
C17	0.0420 (12)	0.0318 (10)	0.0451 (11)	0.0011 (9)	0.0080 (9)	0.0030 (8)
C18	0.0447 (12)	0.0400 (11)	0.0428 (11)	−0.0022 (9)	0.0125 (10)	0.0051 (9)
C19	0.0383 (11)	0.0419 (12)	0.0431 (11)	0.0047 (9)	0.0087 (9)	−0.0020 (9)
C20	0.0565 (14)	0.0310 (10)	0.0501 (12)	0.0049 (10)	0.0125 (11)	0.0030 (9)
C21	0.0484 (13)	0.0362 (11)	0.0421 (11)	−0.0007 (9)	0.0117 (10)	0.0044 (9)
C22	0.0417 (12)	0.0301 (10)	0.0443 (11)	0.0040 (8)	0.0195 (9)	0.0077 (8)
C23	0.0522 (14)	0.0414 (12)	0.0539 (13)	0.0083 (10)	0.0268 (11)	0.0052 (10)
C24	0.0488 (15)	0.0526 (14)	0.091 (2)	0.0154 (12)	0.0369 (15)	0.0208 (14)
C25	0.0443 (15)	0.0544 (15)	0.094 (2)	0.0035 (12)	0.0082 (14)	0.0240 (15)
C26	0.0657 (18)	0.0559 (15)	0.0608 (15)	−0.0032 (13)	−0.0010 (13)	0.0053 (12)
C27	0.0558 (14)	0.0444 (12)	0.0498 (13)	0.0041 (11)	0.0202 (11)	0.0017 (10)

### Geometric parameters (Å, °)

**Table:**

Cl1—C11	1.738 (2)	C11—C12	1.377 (4)
Cl2—C13	1.733 (2)	C12—C13	1.386 (3)
Cl3—C19	1.739 (2)	C12—H12	0.9300
Cl4—C21	1.730 (2)	C14—H14B	0.9700
O1—C7	1.351 (2)	C14—H14A	0.9700
O1—C6	1.449 (2)	C15—C16	1.491 (3)
O2—C7	1.191 (3)	C16—C17	1.395 (3)
O3—C15	1.344 (3)	C16—C21	1.396 (3)
O3—C14	1.457 (3)	C17—C18	1.378 (3)
O4—C15	1.198 (3)	C17—H17	0.9300
N1—N2	1.350 (2)	C18—C19	1.379 (3)
N1—C5	1.352 (3)	C18—H18	0.9300
N1—C22	1.438 (3)	C19—C20	1.376 (3)
N2—N3	1.306 (3)	C20—C21	1.380 (3)
N3—C4	1.360 (3)	C20—H20	0.9300
C4—C5	1.373 (3)	C22—C23	1.378 (3)
C4—C14	1.484 (3)	C22—C27	1.384 (3)
C5—C6	1.492 (3)	C23—C24	1.382 (4)
C6—H6A	0.9700	C23—H23	0.9300
C6—H6B	0.9700	C24—C25	1.368 (4)
C7—C8	1.494 (3)	C24—H24	0.9300
C8—C9	1.394 (3)	C25—C26	1.378 (4)
C8—C13	1.398 (3)	C25—H25	0.9300
C9—C10	1.378 (3)	C26—C27	1.376 (4)
C9—H9	0.9300	C26—H26	0.9300
C10—C11	1.373 (4)	C27—H27	0.9300
C10—H10	0.9300		
			
C7—O1—C6	114.32 (15)	O3—C14—H14A	109.2
C15—O3—C14	115.79 (18)	C4—C14—H14A	109.2
N2—N1—C5	110.66 (17)	H14B—C14—H14A	107.9
N2—N1—C22	119.68 (17)	O4—C15—O3	123.4 (2)
C5—N1—C22	129.66 (17)	O4—C15—C16	125.3 (2)
N3—N2—N1	107.09 (17)	O3—C15—C16	111.32 (18)
N2—N3—C4	109.52 (18)	C17—C16—C21	117.27 (19)
N3—C4—C5	108.01 (19)	C17—C16—C15	120.06 (18)
N3—C4—C14	120.3 (2)	C21—C16—C15	122.52 (19)
C5—C4—C14	131.7 (2)	C18—C17—C16	121.93 (19)
N1—C5—C4	104.71 (18)	C18—C17—H17	119.0
N1—C5—C6	122.59 (19)	C16—C17—H17	119.0
C4—C5—C6	132.6 (2)	C17—C18—C19	118.8 (2)
O1—C6—C5	108.05 (16)	C17—C18—H18	120.6
O1—C6—H6A	110.1	C19—C18—H18	120.6
C5—C6—H6A	110.1	C20—C19—C18	121.1 (2)
O1—C6—H6B	110.1	C20—C19—Cl3	118.39 (17)
C5—C6—H6B	110.1	C18—C19—Cl3	120.48 (17)
H6A—C6—H6B	108.4	C19—C20—C21	119.3 (2)
O2—C7—O1	122.27 (18)	C19—C20—H20	120.3
O2—C7—C8	126.98 (18)	C21—C20—H20	120.3
O1—C7—C8	110.74 (16)	C20—C21—C16	121.4 (2)
C9—C8—C13	117.22 (19)	C20—C21—Cl4	116.42 (16)
C9—C8—C7	119.93 (18)	C16—C21—Cl4	122.19 (17)
C13—C8—C7	122.81 (19)	C23—C22—C27	121.4 (2)
C10—C9—C8	122.2 (2)	C23—C22—N1	119.6 (2)
C10—C9—H9	118.9	C27—C22—N1	119.02 (18)
C8—C9—H9	118.9	C22—C23—C24	118.7 (2)
C11—C10—C9	118.7 (2)	C22—C23—H23	120.7
C11—C10—H10	120.6	C24—C23—H23	120.7
C9—C10—H10	120.6	C25—C24—C23	120.5 (2)
C10—C11—C12	121.6 (2)	C25—C24—H24	119.8
C10—C11—Cl1	119.8 (2)	C23—C24—H24	119.8
C12—C11—Cl1	118.6 (2)	C24—C25—C26	120.4 (3)
C11—C12—C13	119.0 (2)	C24—C25—H25	119.8
C11—C12—H12	120.5	C26—C25—H25	119.8
C13—C12—H12	120.5	C27—C26—C25	120.1 (3)
C12—C13—C8	121.3 (2)	C27—C26—H26	119.9
C12—C13—Cl2	116.37 (17)	C25—C26—H26	119.9
C8—C13—Cl2	122.32 (17)	C26—C27—C22	118.9 (2)
O3—C14—C4	111.88 (18)	C26—C27—H27	120.5
O3—C14—H14B	109.2	C22—C27—H27	120.5
C4—C14—H14B	109.2		

### Hydrogen-bond geometry (Å, °)

**Table:**

*D*—H···*A*	*D*—H	H···*A*	*D*···*A*	*D*—H···*A*
C6—H6B···O4	0.97	2.49	3.280 (3)	139.
C9—H9···N2^i^	0.93	2.58	3.288 (3)	134.

Symmetry codes: (i) −*x*+1, −*y*+1, −*z*+1.
